# Behavioral and metabolic and effects of ABCG4 KO in the APPswe,Ind (J9) mouse model of Alzheimer’s disease

**DOI:** 10.21203/rs.3.rs-3014093/v1

**Published:** 2023-06-08

**Authors:** Vincent Fong, Babunageswararao Kanuri, Owen Traubert, Min Lui, Shailendra B. Patel

**Affiliations:** Department of Internal Medicine, Division of Endocrinology, Diabetes and Metabolism, University of Cincinnati; Department of Internal Medicine, Division of Endocrinology, Diabetes and Metabolism, University of Cincinnati; Department of Internal Medicine, Division of Endocrinology, Diabetes and Metabolism, University of Cincinnati; Department of Pathology & Laboratory Medicine, University of Cincinnati; Department of Internal Medicine, Division of Endocrinology, Diabetes and Metabolism, University of Cincinnati

**Keywords:** Alzheimer&rsquo;s disease, ABCG4, cholesterol metabolism, behavior, glucose metabolism

## Abstract

The pathogenesis of Alzheimer’s disease (AD) is complex and involves an imbalance between production and clearance of amyloid-ß peptides (Aß), resulting in accumulation of Aß in senile plaques. Hypercholesterolemia is a major risk factor for developing AD, with cholesterol shown to accumulate in senile plaques and increase production of Aß. ABCG4 is a member of the ATP-binding cassette transporters predominantly expressed in the CNS, and has been suggested to play a role in cholesterol and Aß efflux from the brain.

In this study, we bred *Abcg4* knockout (KO) with the APP^Swe,Ind^ (J9) mouse model of AD to test the hypothesis that loss of *Abcg4* would exacerbate the AD phenotype. Unexpectedly, no differences were observed in Novel object recognition (NOR) and Novel object placement (NOP) behavioral tests, or on histologic examinations of brain tissues for senile plaque numbers. Furthermore, clearance of radiolabeled Aß from the brains did not differ between *Abcg4* KO and control mice. Metabolic testing by indirect calorimetry, glucose tolerance test (GTT) and insulin tolerance test (ITT), were also mostly similar between groups with only a few mild metabolic differences noted.

Overall these data suggest that the loss of ABCG4 did not exacerbate the AD phenotype.

## Introduction

Alzheimer’s disease (AD) is the most common cause of dementia in people over the age of 65y. It is a progressive neurodegenerative disorder that can manifest as deficits in memory, executive function, visuospatial cognition, language function, and personality changes(Albert et al. 2011; McKhann et al. 2011). While a clinical diagnosis of probable AD dementia can be made if other causes of dementia are ruled out, a definitive diagnosis of AD neuropathological changes requires a histological examination of brain tissue. Key neuropathological features of AD include neurofibrillary tangles (NFTs), primarily composed of abnormally phosphorylated tau protein, extracellular deposition of amyloid-ß peptides(Aß) in senile plaques, and dystrophic neurites(Hyman et al. 2012; Montine et al. 2012).

The amyloid hypothesis of AD postulates that an imbalance between Aß production and clearance, resulting in the accumulation of Aß is a driver of AD pathogenesis(Hardy and Selkoe 2002). While rare genetic early-onset forms of AD are associated with the over-production of abnormal proteins, including Aß and tau(Scheuner et al. 1996; Naj et al. 2017), the more common late-onset Alzheimer’s disease is associated with reduced clearance of Aß from the brain(Mawuenyega et al. 2010; Tarasoff-Conway et al. 2015; Zuroff et al. 2017). However, therapies utilizing monoclonal antibodies targeting Aß to increase efflux from the brain have been largely unsuccessful(Salloway et al. 2014; Doody et al. 2014), indicating that AD pathogenesis is not so simple.

AD is a complex disease, and its etiology is likely multifactorial. There is a growing body of work which links neuroinflammation, oxidative damage, and dysfunctional glucose and lipid metabolism to AD(Kapogiannis and Mattson 2011; Croteau et al. 2018; Butterfield and Halliwell 2019b, a; Behl et al. 2020; Leng and Edison 2020; Ionescu-Tucker and Cotman 2021; Meng et al. 2022). Accordingly, diabetes mellitus and hypercholesterolemia are major risk factors for developing AD(Shepardson et al. 2011a; 2013; Arnold et al. 2018; Meng et al. 2022). Patients with AD have brain insulin resistance(Arnold et al. 2018), and treatment with intranasal insulin improves cognitive function(Reger et al. 2008). Meanwhile, cholesterol has been shown to accumulate in senile plaques(Mori et al. 2001) and increases production of Aß(Shepardson et al. 2011a), but evidence on the effects of statins on cognitive function is mixed(Kurata et al. 2011; Sano et al. 2011; Shepardson et al. 2011b; Tong et al. 2012). Nevertheless, Apolipoprotein E4 remains the most potent genetic risk factor for the development of AD(Naj et al. 2017). Interestingly, of the identified transporters that effl ux Aß out of the brain, many, like LRP1, LRP2, ABCA1, ABCB1 (also known as P-glycoprotein-1), and ABCG4(Shibata et al. 2000; Cirrito et al. 2005; Bell et al. 2007; Do et al. 2012; Dodacki et al. 2017), also have roles in cholesterol metabolism.

ABCG4 is a member of the ATP-binding cassette transporter family that regulates cholesterol homeostasis; ABCG4 is predominantly expressed in the CNS(Yoshikawa et al. 2002; Cserepes et al. 2004; Bojanic et al. 2010). It has also been suggested to have a function in glucose-stimulated insulin secretion (GSIS)(Hou et al. 2016). In the brain, ABCG4 is expressed in neurons, astrocytes, microglia, and capillary endothelial cells at the blood-brain barrier(BBB) (Tarr and Edwards 2008; Uehara et al. 2008; Bojanic et al. 2010; Dodacki et al. 2017). Abcg4 may play a role in cholesterol efflux from the brain(Wang et al. 2004, 2008; Vaughan and Oram 2006), and *in vitro* studies suggest it may also play a role in the export of Aß from the brain at the BBB (Do et al. 2012; Dodacki et al. 2017). Furthermore, in *in vitro* studies, ABCG4 was found to inhibit γ-secretase activity, thus reducing Aß production(Sano et al. 2016). *Abcg4*^−/−^ mice were reported to have a deficit in contextual memory(Bojanic et al. 2010) though no other confirmatory reports have been published. It has therefore been posited that *Abcg4* may play a protective role against the development of AD.

We sought to test the hypothesis that *Abcg4* may be involved in AD pathogenesis using *Abcg4* knockout (KO) mice. We chose the APP^Swe,Ind^ (also referred to as J9) mouse model of AD because it has been reported to have a slower onset AD than other models, and loss of *Abcg4* on this background would be expected to accelerate onset, should the hypothesis be supported. The J9 model is a transgenic mouse that expresses human amyloid precursor protein (APP) with Swedish (K670N/M671L) and Indiana (V717F) mutations, which increases Aß formation and favors Aß42, the form more likely to be found in senile plaques(Hsia et al. 1999; Mucke et al. 2000). We crossed J9 mice with *Abcg4* KO mice and assessed metabolic and behavioral effects.

## Methods

### Animal care

All animal protocols were approved by the University of Cincinnati IACUC, Cincinnati, OH. Mice were group-housed in individually ventilated PIV cages maintained on 14h:10h light and dark cycles and fed a standard chow diet (Envigo 7912; Harlan Teklad, Madison, WI) with access to water *ad libitum* unless otherwise specified by individual experimental protocols.

Tg(PDGFB-APP^SwInd^)J9Lms embryos submitted by Gladstone Institute of Neurological Disease (San Francisco, CA) to Jackson Labs (Bar Harbor, ME) were revived from cryopreservation. Three founder mice were received, and one bred successfully establishing the TgJ9 + mouse line. The *Abcg4* KO mice line was generated as previously described(Dodacki et al. 2017). *Abcg4* KO mice were bred with TgJ9 + mice to produce *Abcg4*^+/−^, J9 + mice. These mice were bred with *Abcg4*^+/−^, J9-mice to produce the experimental cohort and ensure no more than a single copy of the J9 transgene was present in any animal.

### Genotyping details

Genomic DNA was isolated from a tail snip. The genotype of *Abcg4* allele was determined by PCR using primers (5′-CTGCCCTCCCTTATCAATC-3′) and (5′-TATCACAAGCCAGCCTTCTCGG-3′) to detect a 423 bp fragment for the WT allele, and primers (5′-CTGCCCTCCCTTATCAATC-3′) and (5′-TTGCTCACCATGGTGGCGACCGGTGG-3′) primers were used to detect a 400 bp fragment for the mutant allele. The presence of the J9 transgene was determined by amplifying a 360bp fragment using primers (PDAPP-F; GGTGAGTTTGTAAGTGATGCC and PDAPP-R; TCTTCTTCTTCCACCTCAGC3. PCR products were run on a 1.5% agarose gel under standard conditions using a 100bp DNA ladder for size identification. DNA was stained using SYBR Safe DNA gel stain (S33102, Thermo Fisher Scientific, Waltham, MA) and scanned on a gel station (Universal Hood II, Bio-Rad).

### Behavioral testing

Behavioral testing was performed by the University of Cincinnati Mouse Metabolic Phenotyping Center Animal Behavior Core. Mice were assessed at 6–7 months of age and 16–19 months of age.

Open field test: Animals were placed into a novel open field environment (e.g., ~ 36 ×36 in a plastic box or circular field) for up to 30 min. Time spent in the middle and time in the periphery are recorded. Time spent in the periphery near the walls is an indicator of anxiety(Choleris et al. 2001).

Novel object recognition (NOR): Mice were placed into the open field apparatus, as described above, containing 2 different objects. Animals were given 15 min to explore the objects and returned to their home cages. While the mice were away from the arena, one object was replaced with a different object. The animals were returned to the apparatus 24hrs later with one of the former objects and a new object. The difference in the amount of time exploring the new vs. familiar object reflects the memory of the previous experience and the animal’s non-spatial learning(Antunes and Biala 2012).

Novel object placement (NOP): Mice were placed into the open field apparatus, as described above, containing 2 different objects. Animals were given 15 min to explore the objects and returned to their home cages. While the mice were away from the arena, one object was moved to a different location within the apparatus. The animals were returned to the apparatus 24hrs later with the same objects, one object in the same location and one object in a different location. The difference in the amount of time exploring the moved vs. unmoved object reflects the memory of the previous experience and the animals spatial learning(Antunes and Biala 2012).

### Indirect calorimetry

Mice were individually housed in chambers maintained at 23°C with 12h:12h light:dark cycle for simultaneous measurement of oxygen consumption (vO2, ml/h), carbon dioxide production (vCO2, ml/h), energy expenditure, respiratory exchange ratio (RER) and locomotor activity via indirect calorimetry (TSE Systems, Chesterfield, MO, USA). Energy expenditure was calculated using the simplified Weir equation (H = 1.44 (3.94 VO2 + 1.11 VCO2), and energy expenditure or heat (H) was expressed as kcal/h). Previously, the instrument was calibrated with gas cylinders containing nitrogen, 1% carbon dioxide, and atmospheric air mixture (oxygen 20.7%, carbon dioxide 0.03%). Data were acquired every 20 minutes using the LabMaster software (TSE Systems).

### Food Intake

Food intake was assessed with a BioDAQ Food intake monitoring system (Research Diets, Inc., New Brunswick, NJ) between the age 8–12 months and 16–18 months. Mice were individually housed in BioDAQ cages, which monitored the weight of food in the hopper. Water was provided *ad libitum*. Cumulative food intake over 3 days was calculated for each mouse in each trial. For each age group, mice were tested twice with a one-week gap between trials to assess if potential differences in cognitive ability affected the feeding behavior of the mice due to the novel environment of the BioDAQ cage.

### Glucose (GTT) and insulin (ITT) tolerance tests

Mice were fasted for 4–6h and then administered via intraperitoneal injection 2g/kg glucose for GTT or 0.6IU/kg human insulin, Humulin R (HI-213; Lilly, Indianapolis, IN) for ITT. Blood glucose was measured with Accu-Chek Nano electronic glucometer (Roche Applied Science, Indianapolis, IN, USA) at 0, 30, 60, 90, and 120min after intraperitoneal administration of glucose or insulin. If blood glucose dropped below 40mg/dL, the test was terminated, and the animal was administered glucose.

### Measurement of Aß clearance

A guide stainless steel cannula (22-gauge, Plastics One, Roanoke, VA) was implanted stereotaxically into the right caudate putamen of anesthetized mice. The cannula tip coordinates for placement were 0.9 mm anterior from bregma, 1.9 mm lateral from midline, and 2.9 mm below the surface. Animals were allowed to recover for 4–5hto allow for some blood-brain barrier recovery, but before substantial inflammatory response develops. Tracer fluid (1.0μl) containing 50nCi of [^14^C]-inulin (as reference marker) and 50nCi of [^3^H]Aβ_1–42_ was injected with a Motorized Integrated Stereotaxic Injector (iSi) system (Stoelting Co.) into the interstitial fluid (ISF) over 5 min. After injection, the needle was left in place for 5 min. CSF was collected 60min after injection, and brain tissue from the caudate putamen was collected immediately after.

### Thio-S staining for plaques

After completing the experiments, the mice were euthanized by CO_2_ anesthesia followed by thoracotomy to expose the heart. Mice were perfused with chilled PBS followed by 4%PFA via the intracardiac route. The perfused brains were collected and fixed in 4% PFA for another 24hrs. Then transferred to 30% sucrose and stored at 4°C for at least 48hr. Coronal sections (~ 30μm) through the hippocampus were obtained and stained with 0.5% ThioS in 50% EtOH for 10min at room temperature. Sections were rinsed with 50% EtOH, followed by PBS, and mounted on glass slides with aqueous mounting media; then protected from light until visualized by fluorescence microscopy.

## Results

### Presence of Abcg4 did not make a difference in long-term memory.

**Figure 1:**
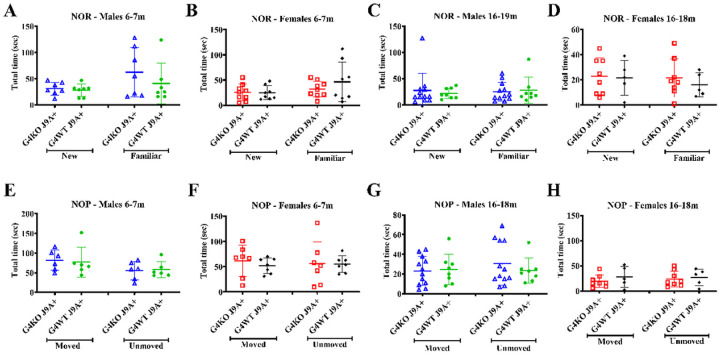
Behavioral testing Novel object recognition (NOR) (A-D) and Novel object placement (NOP) (E-H) tests were performed on *Abcg4*^+/+^J9+ and *Abcg4*^−/−^J9+ mice at age 6–7 months and 16–19 months to assess spatial and non-spatial long-term memory. Total time with each object is shown. Symbols represent individual animals. Lines represents mean +/−1SD.

In order to assess spatial and non-spatial memory, we performed novel object placement (NOP) and novel object recognition (NOR) tests. Animals were tested at 2 timepoints in an attempt to quantify if there was a progressive decline in cognitive function over time. The mice were first tested at 6–7 months of age and then at 16–19 months of age. At both time points, the performance of *Abcg4*^−/−^, J9 + mice did not differ from the performance of the *Abcg4*^−/−^, J9 + mice. In both the NOP and NOR tasks, neither group of mice showed a preference for the novel objects or the moved objects (Fig. 1). Wild-type mice are expected to have a preference for novel objects, suggesting a defect with memory in the J9 + mice, even at the early timepoint, since the animals did not prefer the more familiar object or placement.

### Metabolic effects

**Figure 2:**
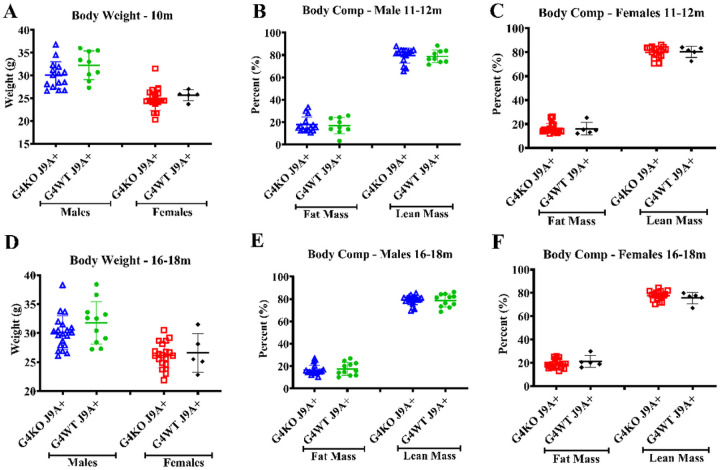
Body weight and composition *Abcg4*^+/+^J9+ and *Abcg4*^−/−^J9 mice were weighed monthly, and body composition was measured by EchoMRI every 2–4 months. Symbols represent individual animals. The error bars represent mean +/−1SD. No statistically significant differences were observed between genotypes.

**Figure 3:**
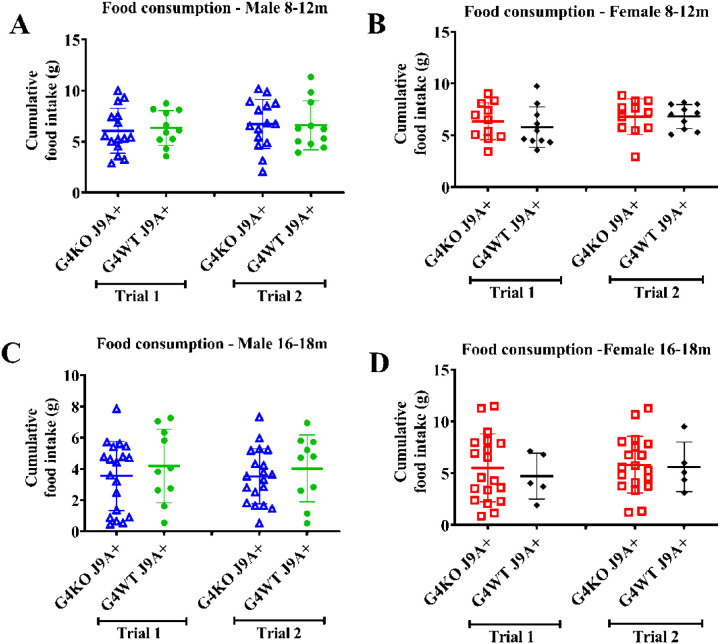
Food Consumption Food consumption of *Abcg4*^+/+^J9+ and *Abcg4*^−/−^J9 mice was assessed with a BioDAQ Food intake monitoring system between ages 8–12 months (A-B) and 16–18 months (C-D). Cumulative food intake over 3 days is displayed for each trial with a one-week gap between trials. Symbols represent individual animals. The error bars represent mean +/−1SD. No statistically significant differences were observed between genotypes or between trials.

**Figure 4:**
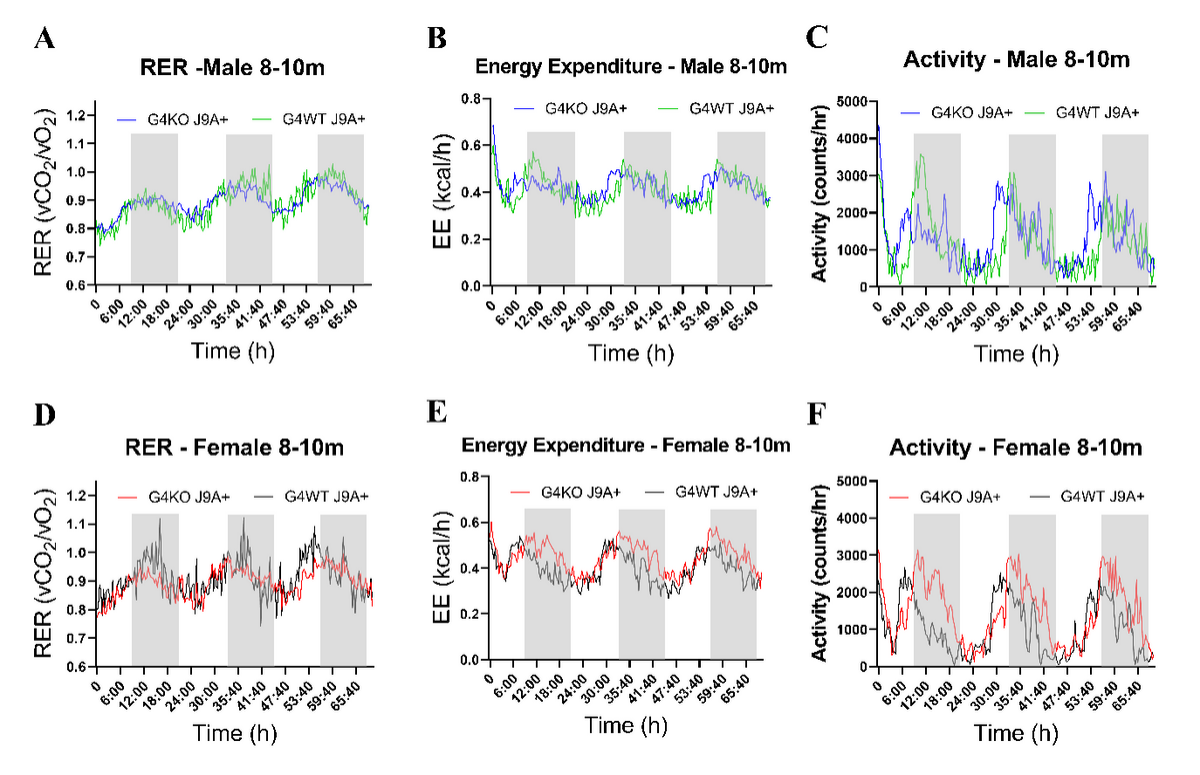
Metabolic profile 8–10 month Metabolic profile of male(A-C) and female(D-F) *Abcg4*^+/+^J9+ (G4WT J9+, n=6 male, 7 female) and *Abcg4*^−/−^J9+ (G4KO J9+, n=11 male, 9 female) mice were assessed by indirect calorimetry between 8–10 months of age. Each line represents the mean of each respective genotype measured every 20min. The shaded area indicates the dark cycle. No consistent patterns to distinguish between the different genotypes were noted.

**Figure 5:**
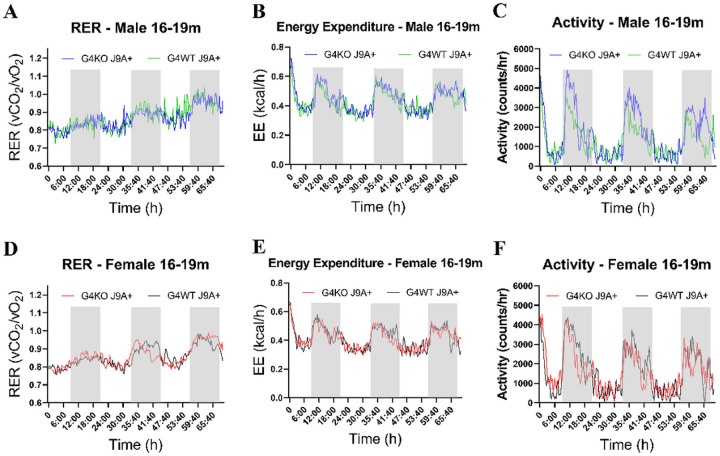
Metabolic profile 16–19 month Metabolic profile of male(A-C) and female(D-E) *Abcg4*^+/+^J9+ (G4WT J9+, n=7 male, 4–5 female) and *Abcg4*^−/−^J9+ (G4KO J9+, n=7 male, 7 female); mice were assessed by indirect calorimetry between 16–19 months of age. Each line represents the mean of each respective genotype measured every 20min. The shaded area indicates the dark cycle.

Despite not finding any differences in behavior, due to the association of AD with metabolic dysfunction, we were also interested in the metabolic effects of *Abcg4* KO and the J9 model. Body weights were measured monthly, and body composition every 2–4 months. Throughout the course of the experiment, both male and female *Abcg4*^−/−^, J9 + mice had similar body weights and body composition as their respective *Abcg4*^+/+^, J9 + counterparts (Fig. 2). The presence or absence of *Abcg4* in the J9 strain (*Abcg4*^+/+^, J9 + vs. *Abcg4*^−/−^, J9+) did not demonstrate any difference in body weight or composition. Similarly, as expected, there was no significant difference in mean food intake, energy expenditure, activity, or respiratory exchange rate (RER) between groups at either time point (Figs. 3–5).

**Figure 6:**
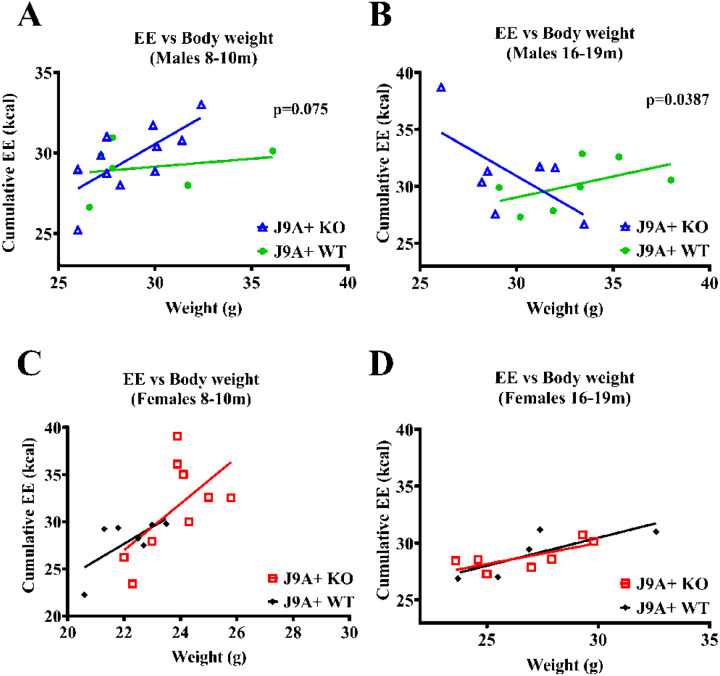
Energy Expenditure vs Body Weight Linear regression of the relationship between cumulative energy expenditure (EE) measured by indirect calorimetry and body weight was calculated for male(A-B) and female(C-D) *Abcg4*^+/+^J9+ and *Abcg4*^−/−^J9 mice. For male mice, a p-value testing the null hypothesis that the slopes are the same is displayed. P-value<0.05 was considered significant. The was no significant difference in the slopes of female mice.

When comparing energy expenditure to body weight, *Abcg4*^−/−^, J9 + and *Abcg*^+/+^, J9 + female mice displayed the expected pattern of increasing EE as body weight increases (Fig. 6C&D). Interestingly, *Abcg4*^−/−^, J9 + male mice also maintained this pattern when younger, however when tested again at 16–19m of age this relationship inverted and demonstrated decreased EE with increased body weight (Fig. 6B). While the average body weights and EE were similar between groups, *Abcg4*^+/+^, J9 + male mice did not demonstrate this inverse relationship (Fig. 6A and 6B).

**Figure 7:**
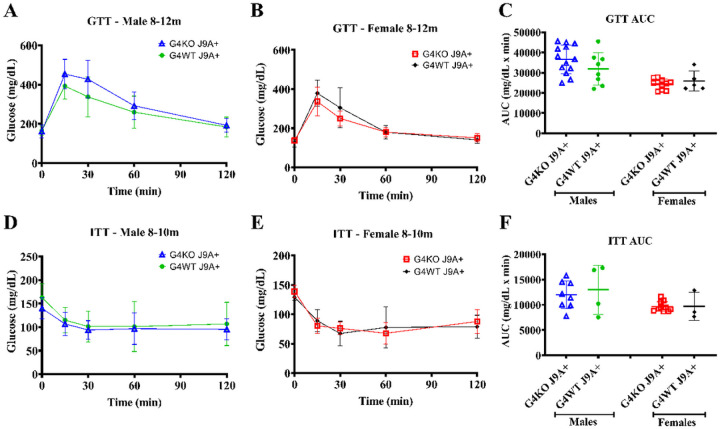
Glucose tolerance and insulin resistance 8–12m Glucose tolerance test (GTT) and insulin tolerance test (ITT) were performed between 8–12 months of age on *Abcg4*^+/+^J9+ and *Abcg4*^−/−^J9+ male and female mice. The error bars denote +/−1SD. Panels C (GTT AUC) and F (ITT AUC) represent the area under the curve (AUC) respective analyses. Symbols represent individual animals. The error bars represent mean +/−1SD

**Figure 8:**
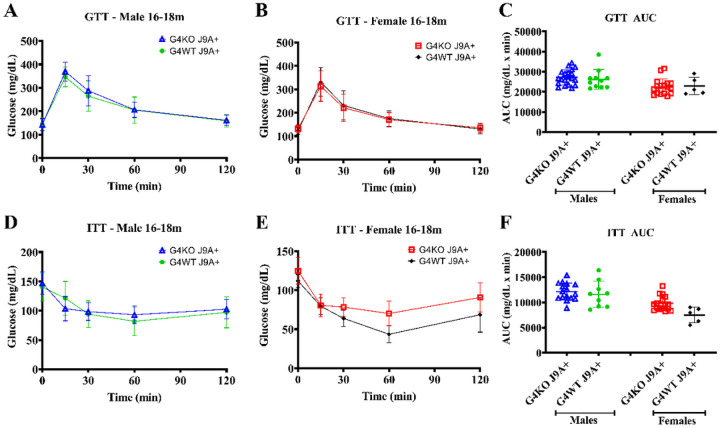
Glucose tolerance and insulin resistance 16–18m Glucose tolerance test (GTT) and insulin tolerance test (ITT) were performed between 16–18 months of age on *Abcg4*^+/+^J9+and *Abcg4*^−/−^J9+ male and female mice. The error bars denote +/−1 SD. Panels C (GTT AUC) and F (ITT AUC) represent the area under the curve (AUC) respective analyses. Symbols represent individual animals. The error bars represent mean +/−1SD

To assess the glucose metabolism, GTT and ITT were performed at 8–12 months and 16–18 months. When compared to male *Abcg4*^+/+^, J9 + mice, male *Abcg4*^−/−^, J9 + mice demonstrated slightly elevated blood glucose levels 30min after glucose injection (Fig. 7A), but not enough to cause statistically significant changes in overall GTT AUC at 8–12 months (Fig. 7C) or at 16–19 months (Fig. 8A). Female *Abcg4*^−/−^, J9 + mice, and *Abcg4*^+/+^, J9 + mice did not show significant differences in glucose tolerance at either timepoints (Figs. 7&8). However, female *Abcg4*^−/−^, J9 + mice did demonstrate reduced insulin sensitivity at the 60 min time-point after insulin injection (Fig. 8E). The biological relevance of this finding is unclear. Male *Abcg4*^−/−^, J9 + mice, and *Abcg4*^+/+^, J9 + mice did not show any difference in insulin sensitivity at either timepoints (Figs. 7 and 8).

### Senile Plaque formation

**Figure 9:**
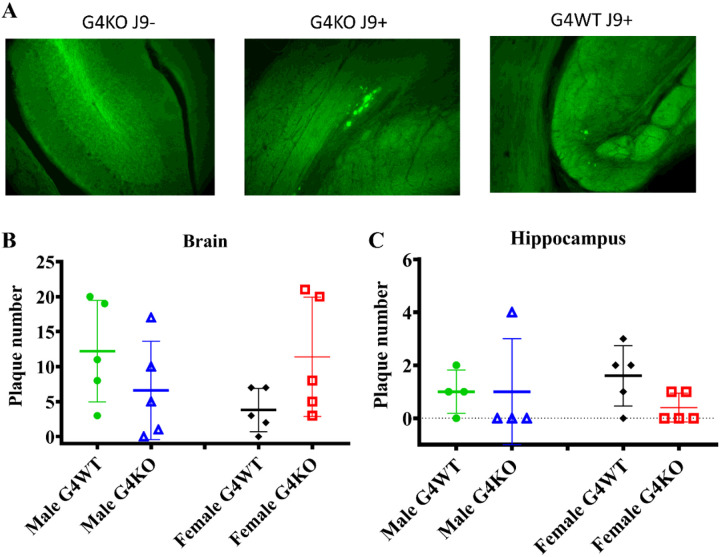
Amyloid plaque formation The brains of *Abcg4*^+/+^J9+ and *Abcg4*^−/−^J9+ mice were stained with ThioflavinS to visualize plaques (A). The number of plaques was counted in whole brain (B) and Hippocampus (C) of *Abcg4*^+/+^J9+ and *Abcg4*^−/−^J9+ mice. Symbols represent individual animals. Lines denote the mean +/−1 SD. (N=5 per group)

After behavioral and metabolic testing was complete, the mice were euthanized, and a subset of the brains were cryopreserved for ThioflavinS staining to visualize amyloid plaques. Plaques were counted in the whole brain and hippocampus to quantify any differences. Plaque numbers varied greatly, but no significant differences in plaque number were noted in whole brain or hippocampi of J9 + mice regardless of the presence or absence of *Abcg4* (Fig. 9).

### Aß clearance

**Figure 10:**
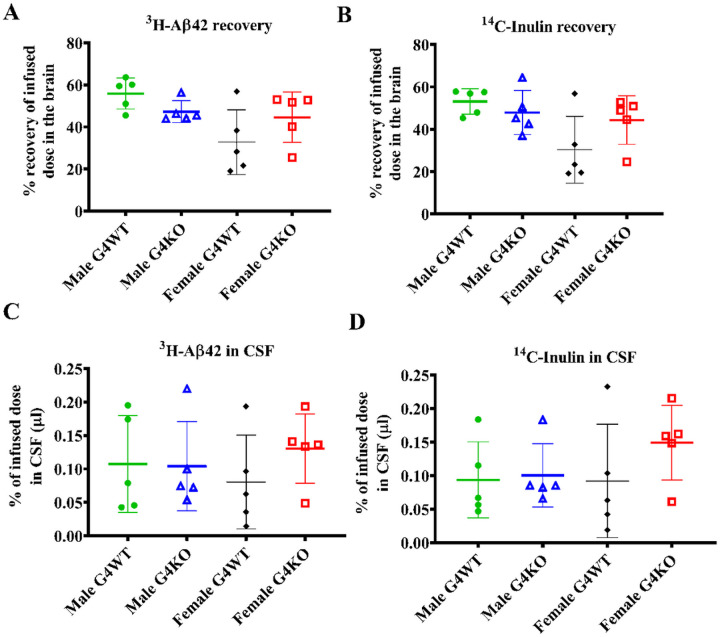
Aβ kinetics The brains of *Abcg4* KO and WT mice were injected with radiolabeled amyloid β protein (Aβ), and the amount remained was measured to assess the Aβ clearance (A). Aβ in CSF was measured to assess passive clearance (C). Radiolabeled inulin, which is cleared only by passive clearance, was measured as a control (B&D). Symbols represent individual animals. Lines denote the mean +/−1 SD.

In light of the lack of difference in AD neuropathologic change with *Abcg4* KO, we directly assessed Aß clearance. Radiolabeled Aß was injected into the brains of *Abcg4* KO and control mice and then measured 60 min later. There was no difference in amount of radiolabeled Aß recovered in the brain and CSF of *Abcg4* KO vs *Abcg4* WT mice (Fig. 10), indicating that Aß clearance rate is unchanged with knockout of *Abcg4*. Radiolabeled inulin, which is cleared only by passive clearance, was injected simultaneously as a control. There was no difference in the amount of radiolabeled inulin recovered in the brain and the CSF of *Abcg4* KO vs. *Abcg4* WT mice either.

## Discussion

We set out to determine the effects of *Abcg4* KO in the J9 model of AD, and unexpectedly did not find any difference in cognitive function or AD-related neuropathologic changes (Figs. 1 and 9). Knockout of *Abcg4* did not change the rate of Aβ clearance (Fig. 10), despite previous studies demonstrating that ABCG4 functions in the efflux of Aβ both in *in vitro* and *in vivo* studies(Do et al. 2012; Dodacki et al. 2017). This suggests that the loss of ABCG4 may have been compensated for by an unidentified mechanism(s), possibly by upregulation of alternate transporters, including its natural binding partner ABCG1(Cserepes et al. 2004). Regardless of these possibilities, loss of Abcg4 alone is not sufficient to accelerate AD pathology. If Abcg4 is still involved in the pathophysiology, it would require additional pathogenic processes, such as defects in cholesterol metabolism. For example, the cholesterol precursor, desmosterol, inhibits Aβ clearance(Dodacki et al. 2017) and is exported from the brain by ABCG4 (Wang et al. 2008; Dodacki et al. 2017). Therefore, the presence of defects that increase the accumulation of desmosterol, like loss of DHCR24(Allen et al. 2019; Kanuri et al. 2021), could potentially make the role of ABCG4 more important. However, these remain speculative at this juncture.

While effects on AD-related behavior and neuropathologic changes were not noted in these studies, there were some mild metabolic changes, mainly some minor differences in glucose and insulin tolerance (Figs. 7 and 8). While it is not clear if these differences are biologically significant or simply Type I error, there are several possible mechanisms by which loss of ABCG4 could have metabolic consequences, including changes in cholesterol homeostasis and defects with GSIS. Although the natural functions of ABCG4 are not completely defined, they include cholesterol and sterol transport. We should keep in mind that the expression of *Abcg4* is not just limited to the CNS. It is also expressed in the retina, hematopoietic cells, spleen, and pancreatic islets(Bojanic et al. 2010; Hou et al. 2016). ABCG4 was discovered to be the target of miR-463–3P, whose expression reduced GSIS(Hou et al. 2016), which suggests that ABCG4 may have a role in GSIS. This mechanism could explain glucose intolerance and insulin resistance in mice with knockout of *Abcg4*. These studies were designed to investigate the effect of the presence or absence of *Abcg4* in the context of the J9 strain (*Abcg4*^+/+^, J9 + vs. *Abcg4*^−/−^, J9+), so it is not possible to draw a conclusion from this data about the individual effects of *Abcg4* KO or J9 transgene alone.

In conclusion, our data show that loss of *Abcg4* did not lead to acceleration of the pathology in a mouse model of Alzheimer’s disease, and that the kinetics of Aß clearance from the brain were not affected by the presence or absence of *Abcg4*.

## Data Availability

Relevant data generated or analyzed during this study are included in this published article; Raw datasets are available from the corresponding author upon reasonable request.
